# Supplementary material for: Multifunctionality of Jasmonic Acid Accumulation during Aphid Infestation in Altering the Plant Physiological Traits That Suppress the Plant Defenses in Wheat Cultivar XN979
Source: Insects. 2023 Jul 11;14(7):622. doi: 10.3390/insects14070622 (PMC10380978; doi:10.3390/insects14070622)
Supplement: Supplementary file 1 [file insects-14-00622-s001.zip › insects-2432741-supplementary.pdf]

Table S1. Primer sequences used for quantification of gene expression by RT-qPCR.

Gene descriptedn	Forward primer sequence (5'-3')	Reverse primer sequence (5'-3')	Tm/°C	References:
TaGAPDH	AACTGTTCATGCCATCACTGCCAC	AGGACATACCAGTGAGCTTGCCAT	53	[26]
TaPAL	GAGATGAGACCCAGAGAGAGTTGACA	AAGGGAGCTGAGATTGCCATGG	55	[26]
TaPR-1	TTCATCATCTGCAGCTACAACC	CGGTACATATATACAGCCGGTCTAA	57	[26]
TaTDC	GCTTTCGGATCAAGGCAAG	CGTGTGTGCAAGATAAGCC	51	[26]
TaAOS	TCTCATAGCAGCCGTCAATC	AAAACACGCACACACATACA	60	[18]
TaLOX	GGCCTTCATCCACACCATCACC	TCGCTGAACCGCTTGAACACCT	60	[18]
TaPR-4	ACACCGTCTTCACCAAGATCGACA	AGCATGGATCAGTCTCAGTGCTCA	60	[11]
TaNPR1	ACTTGGAAAACAGAGTTGCCTTT	GTACTTTCGCCCTAACTCAACTGT	60	[11]
TaWRKY3	TGCGACTAAACTGATGCG	CTTTGCCATTACCGTCGTC	60	[26]
TaPDF1.2	AGCAACAAACACGACCTACTAGC	CCTGTGTCCCGTAATAAAGTAGGA	60	[11]
